# Vacancy Configuration Effects on Oxygen–Carbon Bonding and Carbon Network Reconstruction in Fullerene Nanocages

**DOI:** 10.3390/ma19214433

**Published:** 2026-10-22

**Authors:** Qing Wang, Jing Xu, Wenchang Zhang, Zixi Wang, Surui Cai, Zhikang Zhao

**Affiliations:** 1 School of Special Purpose Vehicle and Emergency Industry, Suizhou Vocational & Technical College, Suizhou 441300, China; 2 School of New Energy, Wanjiang University of Technology, Ma’anshan 243000, China; 18855958790@163.com (W.Z.); 19155474573@163.com (Z.W.); 19055402608@163.com (S.C.);

**Keywords:** fullerene nanocages, vacancy topology, high-temperature oxidation, reactive molecular dynamics, oxygen activation, carbon network reconstruction

## Abstract

ReaxFF molecular dynamics was used to examine oxygen–carbon bonding and carbon network reconstruction of pristine C_60_, monovacancy C_59_, and two specific C_58_ vacancy configurations at 1600-2800 K. Oxidation and oxygen-free conditions each used three independent trajectories and 200 ps production periods. At 2800 K and nominal oxygen loading of 0.5, the normalized C–O bond order sums were 0.04075 ± 0.03532 for V2-adj and 0.17501 ± 0.04520 for V2-sep. Their initial C–C retention fractions were 0.9617 ± 0.0397 and 0.6231 ± 0.1279, respectively. These late-stage differences persisted across bond order thresholds of 0.30–0.40. Matched oxygen-free controls showed substantial thermal reconstruction, especially for V1, demonstrating that connectivity replacement cannot generally be attributed to oxygen attack. The V2-sep mean was 7.85% below an additive two-V1 bonding benchmark, but the difference was smaller than its propagated sampling uncertainty. The results concern the selected configurations and parametrization, whose initial coordination environments differ; they do not establish universal vacancy separation effects or real soot oxidation kinetics.

## 1. Introduction

Soot comprises complex carbonaceous particles formed during the incomplete combustion of hydrocarbon fuels under high-temperature and oxygen-deficient conditions. It is widely found in aero-engines, diesel engines, gas turbines, and industrial combustion devices. Soot not only reduces combustion efficiency and increases radiative heat loss and particulate emissions, but also adversely affects the atmospheric environment and human health [1]. In recent years, with the development of oxygenated fuels, biofuels, and low-carbon combustion technologies, the research focus has gradually shifted from merely reducing soot emissions to regulating soot particle size, aggregation morphology, nanostructure, surface chemical composition, and oxidative reactivity [2]. Previous studies have shown that soot oxidation reactivity is closely related to carbon layer length, curvature, interlayer spacing, and structural order [3], while the oxygen content of the fuel and engine operating conditions can further alter soot particle morphology and carbon layer arrangement [4]. Raman spectroscopic studies have revealed that soot undergoes aromatic layer rearrangement, defect exposure, and changes in structural order during heating and oxidation [5], and that soot with different degrees of maturity also exhibits distinctly different temperature-dependent responses [6]. Meanwhile, soot oxidation rates are jointly influenced by fuel type and initial nanostructure [7], and surface oxygen-containing functional groups, such as hydroxyl, carbonyl, and ether moieties, may participate in the initial steps of oxidation [8]. Thermal oxidation experiments have further demonstrated that the surface chemical composition and internal carbon structure evolve synchronously during oxidation [9]. In situ transmission electron microscopy has directly revealed different reaction modes, including layer-by-layer consumption from the outer surface, internal pore formation, and eventual particle fragmentation [10]. Collectively, these studies indicate that soot oxidation behavior cannot be described by any single macroscopic parameter alone; instead, it depends on which atomic sites are first attacked by oxygen molecules and how the resulting local reactions subsequently propagate and disrupt the entire carbon skeleton.

High-resolution transmission electron microscopy studies have shown that soot generated during actual combustion does not possess an ideal graphitic structure but instead consists of carbon layers with finite lengths, varying orientations, and different degrees of curvature. In some oxygen-rich flames, distinctly curved, closed, and fullerene-like shell structures also appear within soot particles [11]. Combustion experiments using aviation kerosene and bio-jet fuels have indicated that fuel molecular structure can modify primary particle size, carbon-layer curvature, interlayer spacing, and structural order [12]. Studies of the blended combustion of aviation kerosene and ethanol have also found that oxygenated components can simultaneously affect the location of soot formation, particle morphology, and oxidation activity [13]. The addition of n-pentanol alters the aromatic layer arrangement, surface oxygen-containing functional groups, and degree of structural disorder [14]. Furthermore, the effects of n-butanol and 2,5-dimethylfuran on soot formation and surface chemical properties show clear dependence on molecular structure and blending ratio [15]. However, these experimental characterizations mainly provide statistically averaged information or reveal structural differences before and after a reaction, making it difficult to determine directly at which site a given oxygen molecule first forms a C–O bond or how a local defect develops into pore formation, cage opening, and overall fragmentation. Kato et al. compared structural and spectroscopic predictions obtained from ReaxFF simulations with experimental XPS, Raman, and NMR results, demonstrating that atomistic simulations can, to some extent, link the defect structures of carbon materials to experimental characterization signals [16]. In the soot field, ReaxFF reactive molecular dynamics has already been used to investigate polycyclic aromatic hydrocarbon aggregation and nascent soot particle formation [17], the oxidation of polycyclic aromatic hydrocarbons involving nitrogen-containing species [18], the internal structures of nascent soot particles produced by acetylene pyrolysis [19], the evolution of the physical and chemical properties of soot particles [20], and soot precursor formation pathways at different temperatures [21]. These advances indicate that ReaxFF can dynamically describe bond formation and cleavage, thereby providing an atomistic means of investigating the evolution of carbon skeletons from intact states to oxidatively cracked and fragmented states.

Nevertheless, a notable gap remains between existing simulation studies and the scientific questions addressed here. On the one hand, studies of the reactivity and gasification of defective carbon materials have mainly employed planar graphene or coke models. For example, Xiong et al. investigated the effect of vacancy size on the CO_2_ gasification of graphene, showing that defect size alters reaction initiation sites and the removal behavior of carbon atoms [22]. Liang et al. further pointed out that active sites on graphene are continuously generated, reconstructed, and annihilated during H_2_O gasification [23]. Different sp^2^-bond fractions also affect the reactivity and mechanical stability of coke models [24]. On the other hand, existing fullerene simulations have mainly focused on ring structure evolution during the thermal conversion of C_60_ into porous carbon materials [25], the thermal fragmentation of large fullerenes under oxygen-free, high-temperature conditions [26], and the thermal stability and fracture modes of polymeric fullerene networks [27]. The performance of different ReaxFF parameter sets in predicting the structures and energies of graphene, carbon nanotubes, and fullerenes also varies, indicating that the suitability of a force field must be assessed before formal simulations are conducted [28]. In addition, recent studies have analyzed the thermodynamic and mechanical stability of polymeric C_60_ structures [29] and demonstrated that the removal of one carbon atom from C_60_ can produce monovacancy C_59_ isomers with different local reconstructions [30]. Despite these efforts, existing studies have not yet clearly distinguished the independent effects of the number of missing carbon atoms and the spatial arrangement of vacancies on the oxidation of curved, closed carbon skeletons. In particular, adjacent-divacancy C_58_ and separated-divacancy C_58_ have exactly the same number of carbon atoms, yet they may possess different local coordination states, curvature distributions, structural strains, oxygen accessibilities, and crack propagation pathways. Therefore, whether vacancy topology independently controls the oxidation induction period, cage opening site, and fragmentation mode for the same vacancy count remains an unresolved atomistic question.

Based on these considerations, this study employs ReaxFF reactive molecular dynamics to compare the early oxidation response and carbon network reconstruction of pristine I_h_-C_60_, monovacancy C_59_, adjacent-divacancy C_58_, and separated-divacancy C_58_. C_60_ serves as an idealized curved, pentagon-containing, closed carbon network rather than a direct surrogate for real soot. Basic C/O consistency checks precede simulations at 1600, 2200 and 2800 K with three independent trajectories per oxidation condition and a nominal oxygen loading of N(O_2_)/N(C) = 0.5. Oxygen-loading sensitivity is examined for the two divacancy cages at 2800 K. O–O weakening, C–O bonding, retention and replacement of C–C connections, and carbon charge redistribution are used to assess how vacancy arrangement influences the competition between oxygen interaction and carbon network evolution. The global C–O descriptor includes all initial carbon identities; selected structural snapshots additionally distinguish C–O bonding on and outside the main C–C-connected component, without assigning a full carbon oxide yield. The equal composition C_58_ comparison tests vacancy arrangement at fixed vacancy count, with its associated coordination environments, within the selected parametrization and observation window.

## 2. Computational Details

### 2.1. Construction of Fullerene Nanocage Models

A C_60_ fullerene with ideal I_h_ symmetry was constructed based on the vertices of a truncated icosahedron. The initial carbon cage had an average radius of 3.5188 Å and a nominal C–C bond length of 1.4200 Å. Structural examination confirmed that all 60 carbon atoms in C_60_ were three coordinated.

**Figure 1:**
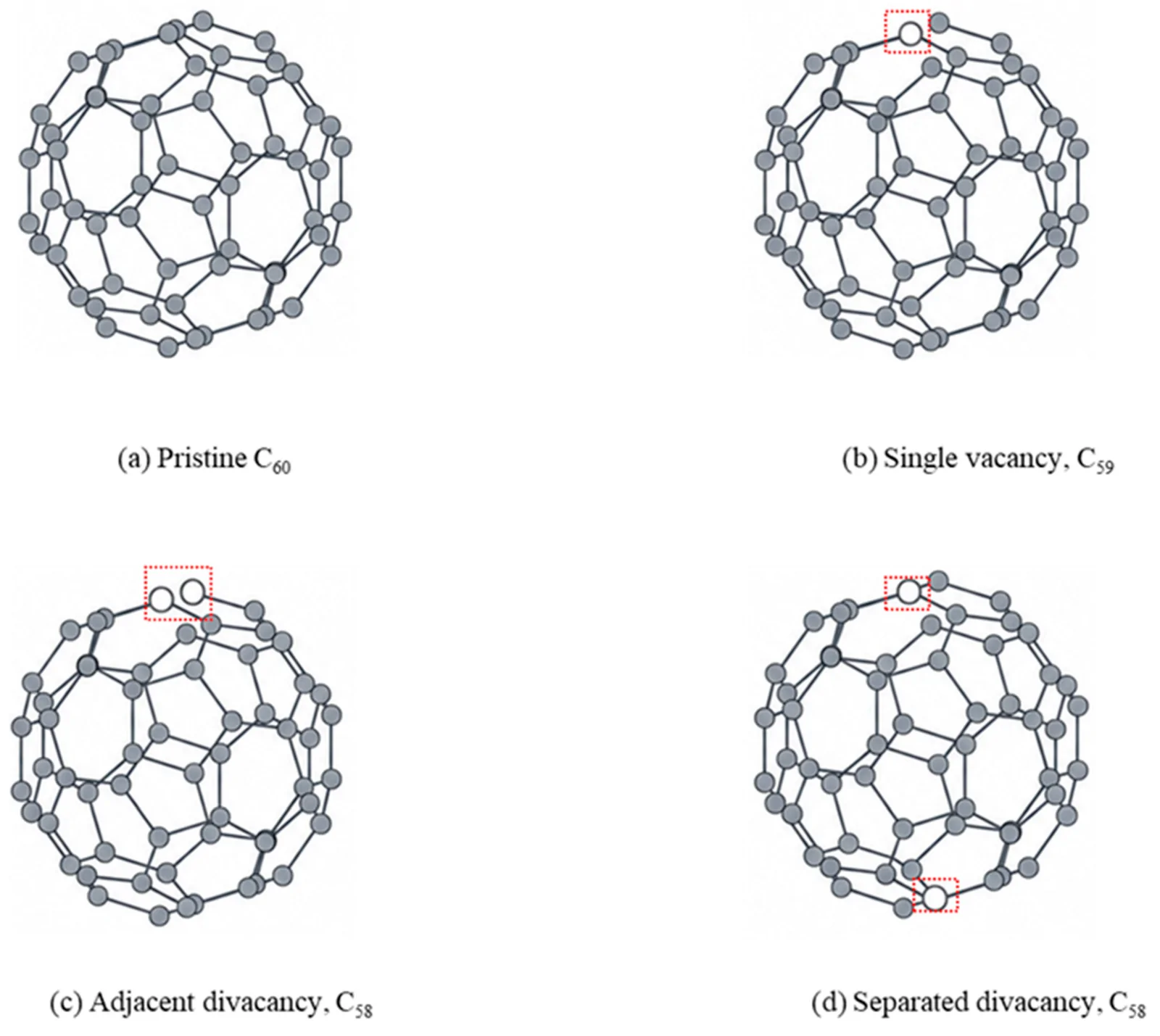
Schematic representations of the initial fullerene nanocage models and vacancy topologies used in the simulations. The red dashed boxes highlight the vacancy sites, and the open circles indicate the original positions of the removed carbon atoms.

Using the same intact C_60_ structure as the parent structure, four fullerene nanocage models were constructed, as shown in Figure 1: pristine C_60_, single-vacancy C_59_, adjacent-divacancy C_58_, and separated-divacancy C_58_. For clarity in the subsequent discussion, the single-vacancy structure is denoted as V1, the adjacent-divacancy structure as V2-adj, and the separated-divacancy structure as V2-sep.

For V2-adj, the two carbon atoms removed from pristine C_60_ were directly bonded, corresponding to a graph distance of 1. For V2-sep, the two removed carbon atoms had a graph distance of 9 and an initial Euclidean distance of 7.0376 Å. The initial numbers of undercoordinated carbon atoms in pristine C_60_, V1, V2-adj, and V2-sep were 0, 3, 4, and 6, respectively.

V2-adj and V2-sep were selected as contrasting vacancy arrangements at equal C_58_ composition and vacancy count, rather than as an exhaustive set of equilibrium isomers. The removed sites are bonded in V2-adj and widely separated in V2-sep. The initial undercoordination nevertheless differs: four carbon atoms in V2-adj and six in V2-sep, compared with three in V1. Thus, this is a comparison of vacancy configuration and its associated local coordination environment, not an isolated test of vacancy separation. No intermediate arrangements or reconstructed isomer ensemble was simulated, and no relative relaxed energies or local strain measures are reported. Neither model is asserted to be the most stable or most frequently observed divacancy. Other arrangements need not show responses intermediate between these two cases because their ring environments, strain and reconstruction pathways can differ. The results therefore demonstrate differences between the selected C_58_ configurations without establishing a general adjacent-versus-separated classification.

No artificial positional or bonding constraints were imposed on the carbon atoms surrounding the vacancies. Therefore, the defective carbon cages were allowed to relax and reconstruct naturally during energy minimization, initial equilibration, heating, and oxidation. Each carbon cage was placed at the center of a three-dimensional periodic cubic simulation cell. A minimum distance of 12 Å was maintained between the initial outer surface of the carbon cage and each boundary of the simulation cell, and O_2_ molecules were randomly distributed around the cage.

### 2.2. Reactive Molecular Dynamics Simulations

All atomistic simulations were performed using the LAMMPS molecular dynamics package(version 22 July 2025) [31]. Chemical bond formation, bond dissociation, and molecular structural rearrangement were described using the ReaxFF reactive force field originally developed by van Duin et al. [32].

The C/H/O parameter set developed by Chenoweth et al. was employed in this study [33]. This parameter set was originally developed for high-temperature hydrocarbon oxidation and includes the C–C, C–O, and O–O interactions required for the carbon–oxygen systems investigated here.

Unlike conventional force fields with fixed molecular connectivity, ReaxFF continuously calculates interatomic bond orders based on instantaneous interatomic distances. Therefore, C–C and O–O bonds can dissociate and new C–O bonds can form naturally during the simulations without prescribing reaction pathways in advance [32,34].

The charge equilibration method was used to calculate the dynamic variation in atomic partial charges during the reactions [35]. The atomic charges were updated at every molecular dynamics time step. The charge equilibration convergence tolerance was set to 1.0 × 10^−6^, the upper cutoff radius was set to 10 Å, and the maximum number of charge iterations was set to 500. The associated numerical implementation followed the reactive molecular dynamics algorithm proposed by Aktulga et al. [36]. QEq charges are model-dependent partial charges, not measured electron populations. In particular, QEq can permit unphysical charge transfer between weakly interacting or dissociating regions [37]. ACKS2 offers a means to reduce this artifact, while eReaxFF incorporates explicit electron degrees of freedom and has been combined with ACKS2 [38]. Neither method was tested here. Changing the charge model also changes electrostatic energies and forces and requires compatible parameters and renewed validation; it cannot be assessed by relabeling the existing QEq charges.

Periodic boundary conditions were applied in all three spatial directions. The molecular dynamics integration time step was set to 0.1 fs. The system temperature was controlled using a Nosé–Hoover thermostat in the canonical NVT ensemble, with a temperature damping parameter of 100 fs.

Following energy minimization, each simulation in the main oxidation matrix consisted of three consecutive stages:

(1) A 10 ps initial equilibration stage at 300 K;

(2) A 20 ps heating stage from 300 K to the target temperature;

(3) A 200 ps production stage at the target temperature.

The target temperatures were 1600, 2200, and 2800 K. These temperatures are higher than those typically used in macroscopic fullerene oxidation experiments. The elevated temperatures were employed to accelerate relatively rare events, including O_2_ activation, C–C bond dissociation, carbon cage reconstruction, and carbon atom removal, so that these processes could be observed within computationally accessible molecular dynamics timescales. Similar accelerated reactive molecular dynamics strategies have been used to investigate the oxidation and evaporation of carbonaceous particles [39].

The elevated temperatures provide accelerated access to reactive events within a 200 ps production window; they do not establish a direct mapping to experimental temperature or time. At lower temperatures, oxygen attachment and carbon network reconstruction may both become slower, but not by the same factor. Their competition can therefore change, and neither the numerical oxidation response nor the V2-adj/V2-sep ranking is assumed to remain unchanged outside 1600–2800 K. The transferable hypothesis is that vacancy arrangement influences the availability and evolution of reactive carbon sites. In particular, the lower late-stage C–O bonding of V2-adj at 2800 K is a condition-specific observation, not a prediction of improved oxidation resistance at all higher temperatures. Lower-temperature calculations over longer observation times and experiments on characterized defect populations would be required to test this extrapolation. Furthermore, a single cage in a periodic oxygen-containing cell does not include the intermolecular environment, agglomerate morphology, or transport effects of bulk fullerene samples [40,41].

### 2.3. Simulation Matrix

The final matrix comprised 84 independent trajectories: 36 principal oxidation trajectories, 36 matched oxygen-free controls and 12 oxygen-loading sensitivity trajectories. Extending an existing trajectory does not create an additional independent replicate.

The principal oxidation matrix included four cage structures, three temperatures and three independent initial-velocity seeds at a nominal oxygen loading of 0.5:

The oxygen-loading labels denote nominal molecule/carbon ratios used in constructing the inputs. The verified principal inputs contain 30 O_2_ molecules for C_60_, 30 for C_59_, and 29 for each C_58_ configuration; the actual N(O_2_)/N_C,0_ ratios are therefore 0.50000, 0.50847 and 0.50000, respectively. For the C_58_ loading tests, nominal loading of 0.25 corresponds to 14 O_2_ molecules and an actual ratio of 0.24138, whereas nominal loading of 1.0 corresponds to 58 O_2_ molecules and a ratio of 1.00000. Thus, V2-adj and V2-sep are matched in composition and actual oxygen loading at each loading label; comparisons across carbon counts are nominally matched. The nominal labels are retained throughout the figures and tables, with exact molecule counts stated here.

4 Cage Structures × 3 Temperatures × 3 Random Seeds = 36 Cases.

The matched oxygen-free matrix included the same four structures and three temperatures, with three independent initial-velocity seeds per condition:

4 Cage Structures × 3 Temperatures × 3 Random Seeds = 36 Control Trajectories.

The oxygen-loading sensitivity matrix focused on the oxidation behavior of the two divacancy structures at 2800 K. Two additional nominal oxygen loadings, N(O_2_)/N(C) = 0.25 and 1.0, were considered, with three independent random seeds used for each condition:

2 Cage Structures × 2 Oxygen Loadings × 3 Random Seeds = 12 Cases.

The detailed simulation conditions are listed in Table 1.

The principal oxidation and loading tests used seeds 1001, 2002 and 3003; the oxygen-free controls used seeds 9001, 9002 and 9003. All final trajectories contained 10 ps equilibration, 20 ps heating and 200 ps production at an integration time step of 0.1 fs. Oxidation and control seeds were independent, so the comparisons were not treated as paired samples.

Continuous late-stage descriptors are reported as mean ± sample SD across three independent trajectories per condition. Table 2 includes the six oxidation descriptors, and Table 3 compares matched controls at 2800 K. Lower-temperature control examples and threshold tests are reported in Section 3.4. Three trajectories give limited sampling of stochastic encounters; mean trends and SD values are not taken as a general demonstration of statistical significance.

The original oxygen-free seed 9001 trajectories were extended from 100 to 200 ps production using their final restart states, without recreating velocities or minimizing again. Two additional seeds, 9002 and 9003, were independently initialized for each condition and followed the same equilibration, heating and 200 ps production protocol. The selected force field file was unchanged. Original and extension segments were combined with duplicate boundary frames removed and uniform 0.1 ps sampling checked. Each final control spanned 0–230 ps with 2301 saved frames; comparison with oxidation used the common 220–230 ps window.

### 2.4. Species Identification and Structural Evolution Analysis

Atomic coordinates, reactive bond orders and molecular species records were saved every 1000 integration steps, corresponding to 0.1 ps. The primary connectivity descriptors use reported pairs with BO ≥ 0.30. Molecular species were identified from the species output together with the bond network. Production-stage persistent connection states were evaluated from unsmoothed records: a state must span the specified elapsed time, with no intervening frame failing the state condition. A persistent state describes the existence of at least one qualifying connection and is not the lifetime of a particular atom pair. States already present at the 30 ps production boundary do not establish a physical reaction onset at 30 ps.

Bond order information can be used to analyze connectivity in reactive MD trajectories [42]. Here, BO = 0.30 defines a connection operationally, and its sensitivity is examined using thresholds of 0.35 and 0.40. A 1.0 ps persistence criterion requires ten consecutive 0.1 ps intervals, hence at least eleven saved frames. Its implementation was corrected to use the actual first-to-last time span rather than a count of ten frames. Sensitivity tests reanalyzed the 48 oxidation trajectories with BO thresholds of 0.30, 0.35 and 0.40 and persistence criteria of 0.5, 1.0 and 2.0 ps. Initial reference C–C and O–O pairs were held fixed. Because the bond output omits connections below BO = 0.30, lower cutoffs cannot be reconstructed from these records. The new matched controls are reported using the primary BO = 0.30 definition. The reported conclusions use these bond order descriptors; geometric cage opening and fragmentation thresholds are not used to establish the mechanisms discussed here.

O_2_ activation was characterized by tracking the O-O bonds belonging to the initially introduced O_2_ molecules. Let *S*_OO_(0) be their initial total bond order and *S*_OO_(*t*) the corresponding value at time *t*. The O-O bond-weakening index was defined as

*W*_OO_(*t*) = 1 − *S*_OO_(*t*)/*S*_OO_(0)

An increase in WOO therefore represents progressive weakening of the initial O-O bonds. Oxygen–carbon bonding was characterized using the normalized global C–O bond order sum,

*S*_CO_^norm^(*t*) = *S*_CO_(*t*)/*N*_C,0_

Here, S_CO_(t) sums all reported C–O connections at BO ≥ 0.30 over the initially introduced carbon atoms, and N_C,0_ is the initial carbon count. Carbon identities remain included if they cease to belong to the main C–C-connected network. Thus, S_CO,norm_ measures global C–O bonding across carbon structures, not exclusively oxygen retained on the surviving cage. Selected snapshots separate contributions from carbon atoms inside and outside the largest C–C-connected component; this frame-level partition is not a late-window average or a detached-product yield. Mean absolute carbon charge likewise averages over all initial carbon identities.

Evolution of the carbon framework is described using the initial C-C bond retention fraction, the number of newly formed C-C bonds and the normalized C-C bond order sum. The retention fraction is defined as

*f*_CC_^ret^(*t*) = *N*_CC_^ret^(*t*)/*N*_CC,0_

where *N*_CC,0_ is the number of initial C-C bonds, and *N*_CC_^ret^(*t*) is the number remaining above BO = 0.30 at time t. C-C connections absent from the initial network but subsequently reaching BO ≥ 0.30 were classified as newly formed C-C bonds. The quantity *S*_CC_(*t*)/*N*_C,0_ was additionally used to characterize the overall C-C bonding strength. Dynamic charge redistribution accompanying oxygen attachment was assessed using the mean absolute partial charge of the carbon atoms.

Initial reference C–C and O–O pairs for the trajectory descriptors are taken from the first saved frame at total time 0 ps, after the initial energy minimization and before equilibration. They need not be identical to the unrelaxed construction-stage topology. Each trajectory uses its own reference for retention, whereas the same reference is held fixed across alternative BO thresholds within that trajectory.

A nominal 1.0 ps moving average is used only for displaying continuous trends. Quantitative late-stage statistics use unsmoothed descriptors. For each trajectory r, X_r_ is the separate average of the 101 saved frames over 220–230 ps, corresponding to production time 190–200 ps. The condition mean is $\bar{X}$ = Σ_r_ X_r_/3 and the sample SD is s_X_ = [Σ_r_(X_r_ − $\bar{X}$)^2^/(3 − 1)]^½^. SEM = s_X_/√3, whereas the manuscript tables report SD. The independent unit is a trajectory, not a saved frame or an atom; in particular, spatial variation in atomic charges does not replace uncertainty in the trajectory-level mean absolute carbon charge. Event status results use the tested persistent-state definitions summarized in Section 3.4.

Initial undercoordination denotes fewer than three carbon neighbors in the constructed cage topology before equilibration, as summarized in Section 2.1. These construction-stage counts characterize the selected models; they are not used as a cage-opening criterion. Loss of an initial C–C connection or formation of a new connection does not by itself demonstrate an open cage, fragmentation or carbon removal.

The reported analyses quantify oxygen–carbon bonding, connection replacement and charge redistribution. The aggregate persistent-state times are operational descriptors of the production-stage bond network. A separate local analysis below follows individual atom pairs at the saved sampling interval; its observed episode spans are not kinetic bond lifetimes. No cage opening distribution, geometric fragmentation threshold, radius-of-gyration evolution or detached CO/CO_2_ yield is used to support the conclusions. Such later-stage assignments would require separate species, spatial and structural evidence.

Local reconstruction was additionally examined in 18 trajectories: V2-adj oxidation at all three temperatures, V2-sep oxidation at 2800 K, and both C_58_ oxygen-free controls at 2800 K, each with three seeds. A fixed construction zone contains the initially undercoordinated carbon atoms and their one-hop neighbors, identified at 1.90 Å in the unrelaxed construction model (11 atoms for V2-adj and 18 for V2-sep). A connection is zone-associated when at least one carbon endpoint belongs to this set; this tracks atom identities, not instantaneous spatial localization. New C–C pairs are absent from the first saved, post-minimization reference. Individual-pair episodes require that same pair to remain above BO = 0.30 in consecutive saved frames; a 1 ps observed span requires eleven 0.1 ps frames. Episodes ending at 230 ps are right-boundary censored. Total time 0–10 ps is equilibration, 10–30 ps is heating and 30–230 ps is production. Fixed-time snapshots were extracted at total times of 0, 30 and 230 ps for all three V2-adj oxygen-containing trajectories with a production temperature of 2800 K. Only the largest C–C-connected component and oxygen directly bonded to its carbon atoms are displayed, with the component size stated in each panel. The saved coordinates are centered and rigidly aligned with a common spatial scale; no optimization or interpolation is applied. Unbound oxygen is omitted. Fixed-pair timings and the numerical local analysis are reported in Section 3.2.

### 2.5. Validation and Consistency Checks of the Computational Protocol

Isolated O_2_, CO and CO_2_ molecules were optimized with the force field used in the production calculations. The optimized O–O distance in O_2_ was 1.2647 Å, the C–O distance in CO was 1.1233 Å, and both C–O distances in linear CO_2_ were 1.2487 Å, with an O–C–O angle of 180.0°. The experimental reference distances tabulated by NIST are 1.2075 Å for O_2_ and 1.1621 Å for CO_2_ [43]. The calculated distances therefore exceed these references by 0.0572 Å (4.74%) and 0.0866 Å (7.45%), respectively. This discrepancy is appreciable, particularly for CO_2_, and the geometries are treated as a limited consistency check rather than quantitative validation. Because these are optimized isolated-molecule geometries, the discrepancy cannot be explained as thermal expansion at the production temperatures.

ReaxFF equilibrium distances arise from an empirical energy expression fitted to multiple quantum mechanical targets; a parametrization intended to describe reaction energetics need not reproduce every experimental molecular distance exactly [33]. This explains why a mismatch is possible, but identifying its specific fitting origin would require an audit of the training targets and parameter optimization. Different parametrizations can change equilibrium structures as well as adsorption and reconstruction energetics; comparisons for sp^2^ carbon illustrate such structural dependence [28]. No alternative parametrization was optimized or tested for O_2_ or CO_2_ in this work, so its effect on these particular bond lengths, or on the vacancy topology ranking, cannot be quantified here.

The oxidation of CO was further evaluated through

CO + 1/2 O_2_ → CO_2_,

with the potential energy change defined as

Δ*E* = *E*_CO2_ − *E*_CO_ − 1/2 *E*_O2_.

The calculated value was −74.890 kcal mol^−1^, reproducing the expected exothermic direction of CO oxidation. This calculation was used as a consistency check of the C-O and O-O energetic description rather than as a high-accuracy prediction of the reaction enthalpy.

The initial C_60_ structure was independently examined before introducing vacancies and molecular oxygen. All 60 carbon atoms were three coordinated, with a mean cage radius of 3.5188 Å and a nominal C-C bond length of 1.4200 Å. A short 1 ps test simulation was subsequently performed to verify the numerical stability of the molecular dynamics integration, charge equilibration procedure and reactive force-field implementation. The calculation was completed normally without numerical instability.

The validation above establishes basic numerical and chemical consistency, but not defect-specific predictive accuracy. The Chenoweth C/H/O parametrization was optimized against a quantum mechanical training set containing oxidation-relevant reactions and transition states, and its published tests addressed hydrocarbon/O_2_ systems [33]. This provides a methodological basis for following coupled C–C, C–O and O–O bond changes, but the original hydrocarbon tests do not establish the accuracy of O_2_ adsorption or dissociation at C_58_ vacancy sites. Likewise, the small-molecule geometries, exothermic CO-oxidation energy and successful integration test reported here cannot validate vacancy reconstruction energies, oxygen-binding energies or reaction barriers. Force-field-dependent behavior in sp^2^ carbon systems has been documented [28]. Using the same potential for all four cages makes the comparisons internally consistent, but does not guarantee cancellation of errors between different defect environments. The present results are therefore interpreted as mechanistic trends within this parametrization. A quantitative assessment would require independent electronic structure benchmarks for relaxed V2-adj/V2-sep geometries and relative energies, molecular and dissociative oxygen adsorption, and competing oxygen attachment and C–C reconstruction barriers; such benchmarks were not performed in the present study.

## 3. Results and Discussion

### 3.1. Temperature-Dependent O_2_ Activation and C–O Bond Formation

**Figure 2:**
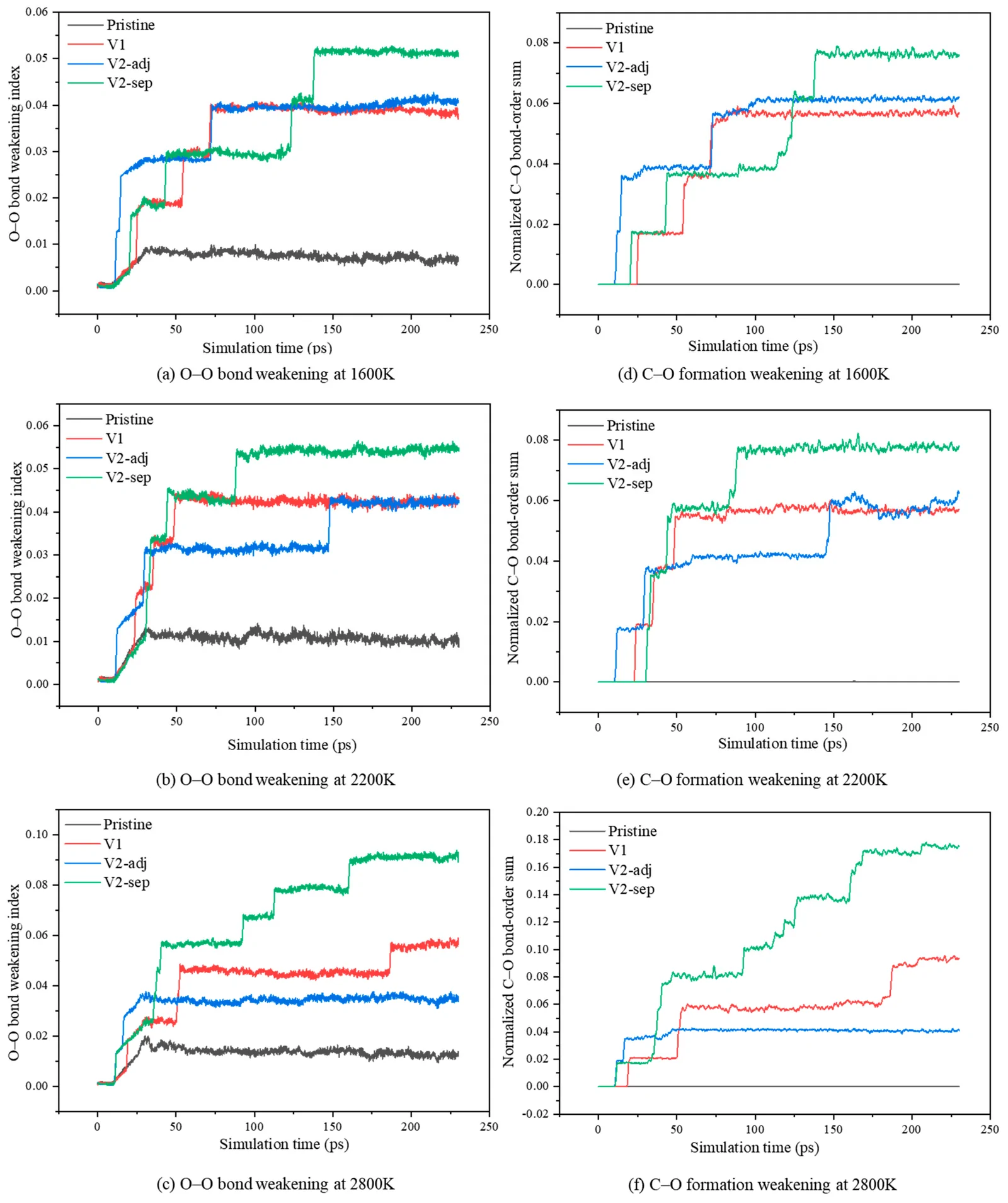
Temperature-dependent O–O bond weakening and C–O bond formation in pristine and defective fullerene nanocages.

Figure 2 compares the O–O bond-weakening index W_OO_ and normalized C–O bond-order sum S_CO,norm_ at nominal loading of 0.5. Displayed curves represent averages of three trajectories with a nominal 1.0 ps moving average. The quantitative values below are recalculated from separate unsmoothed 220–230 ps means for each seed; Table 2 gives their means and sample SDs.

At 1600 K, the mean W_OO_ values were 0.0068 for C_60_, 0.0382 for V1, 0.0408 for V2-adj and 0.0511 for V2-sep. Their mean S_CO,norm_ values were 0, 0.0571, 0.0614 and 0.0762, respectively. The sampled defective cages developed greater O–O weakening and C–O bonding than pristine C_60_ under this condition. These descriptors characterize bonding extent within the observation window, rather than an oxidation rate.

At 2200 K, pristine C_60_ had mean W_OO_ = 0.0101 and no C–O bonding in the late-stage window. Mean S_CO,norm_ values were 0.0570 for V1, 0.0599 for V2-adj and 0.0777 for V2-sep. The increase in pristine W_OO_ therefore did not coincide with late-stage C–O attachment. Table 2 and the individual-seed data show the accompanying variability.

At 2800 K, pristine C_60_ retained a very small late-stage S_CO,norm_ of (1.81 ± 3.13) × 10^−5^. V1 reached S_CO,norm_ = 0.09334 ± 0.03989 and W_OO_ = 0.05680 ± 0.02041. Its mean C–O bonding was higher than at 1600 K, but the stochastic spread remains substantial.

For V2-adj, S_CO,norm_ was 0.06142 ± 0.00320 at 1600 K, 0.05987 ± 0.00509 at 2200 K and 0.04075 ± 0.03532 at 2800 K. The 2800 K mean is lower by 33.7% and 31.9% than the lower-temperature means, but its dispersion is much larger; seed 2002 has zero late-stage C–O bonding. This is a nonmonotonic sampled mean, not a statistically established temperature reversal or a measured elementary rate. Connectivity replacement and thermal controls are examined below before interpreting a possible rebonding contribution.

V2-sep reached W_OO_ = 0.09119 ± 0.01920 and S_CO,norm_ = 0.17501 ± 0.04520 at 2800 K. Its mean C–O bonding was 1.88 times the V1 mean and 4.29 times the V2-adj mean. The three V2-sep S_CO,norm_ values span 0.14293–0.22671, whereas V2-adj spans 0–0.06238; these sampled ranges do not overlap. This supports the direction of the configuration difference in the present sample, without establishing a universal separation effect or a reaction probability per vacancy. Undercoordination and strain can influence adsorption in defective carbon [44,45].

The curves in Figure 2 exhibit stepwise changes and plateaus, indicating intermittent evolution of the mean bonding descriptors. These changes do not uniquely identify individual collision or reaction events; event assignment requires the corresponding atom-resolved histories. Experimental fullerene oxidation involves different sample environments and observation scales [40,41], as discussed in Section 3.4.

S_CO,norm_ measures global C–O bonding over the initial carbon population. It does not separate oxygen retained on the main carbon network from oxygenated carbon outside it. The selected-frame partition in Section 3.2 clarifies this distinction. Detached CO or CO_2_ yields would require a separate complete trajectory-level molecular species and structural analysis; they are not inferred from this global sum.

### 3.2. Carbon Network Reconstruction and Charge Redistribution at 2800 K

**Figure 3:**
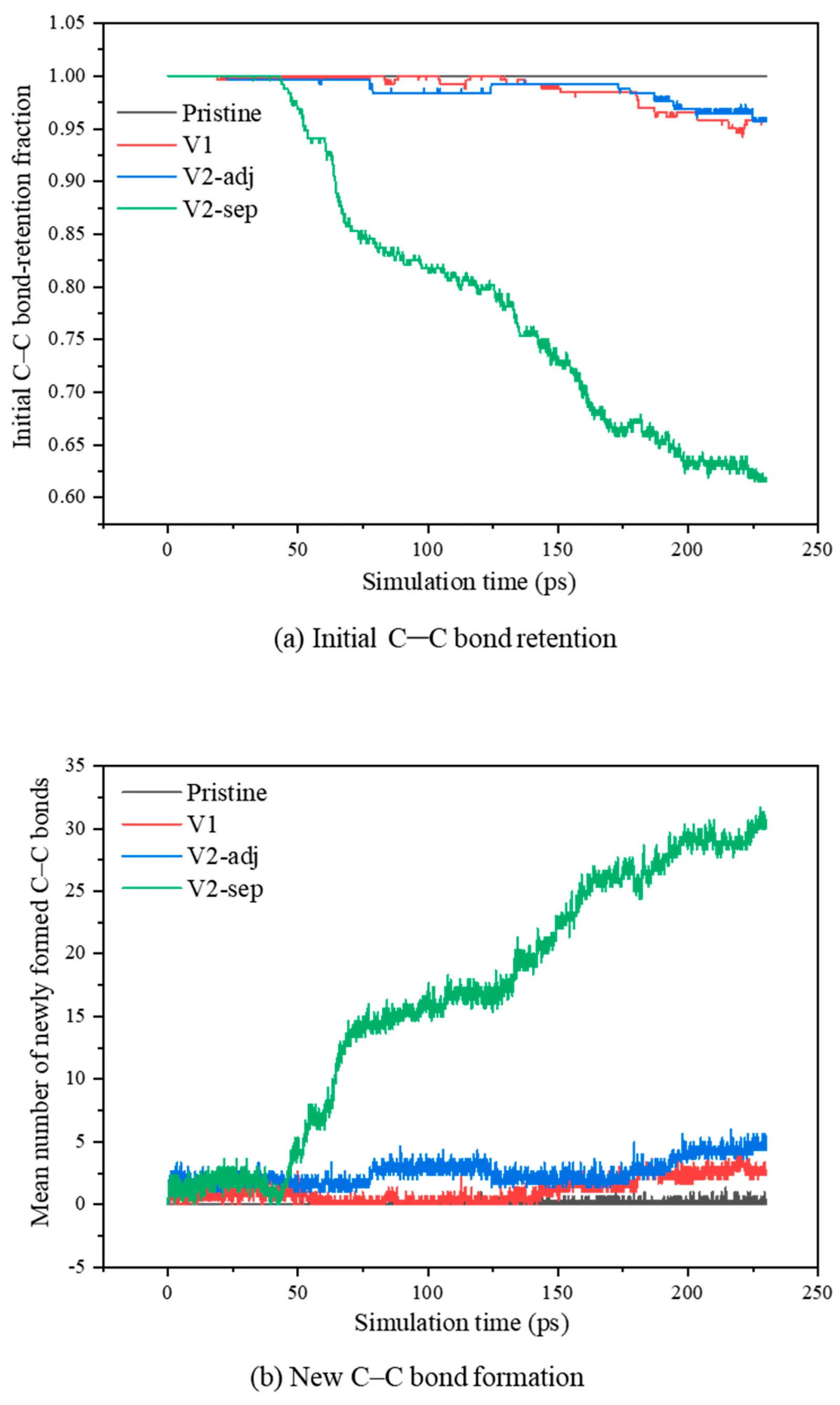

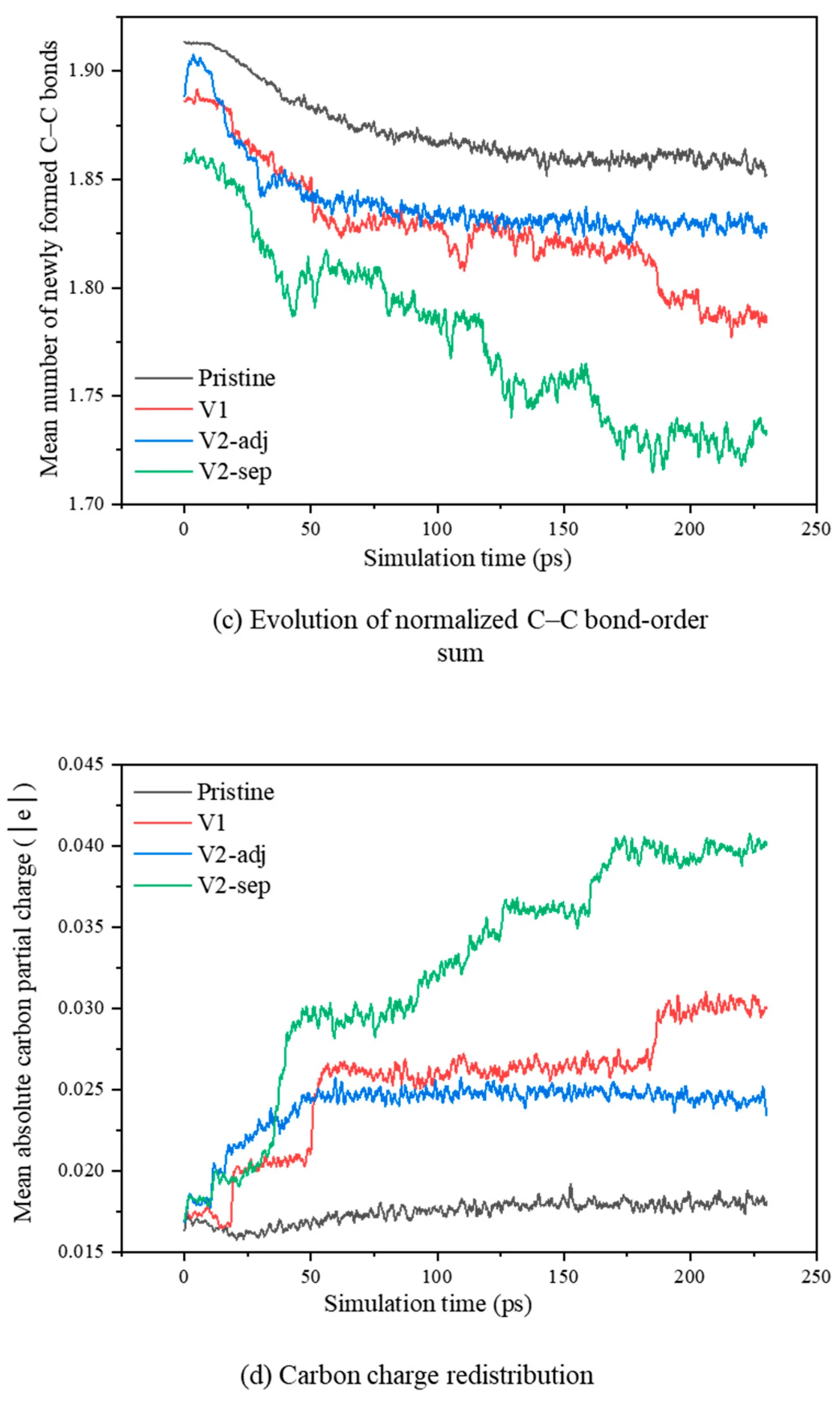
Carbon network reconstruction and charge redistribution of pristine and defective fullerene nanocages at 2800 K.

Figure 3 and Table 2 combine initial C–C retention, new C–C connections, normalized C–C bond order sum and mean absolute carbon charge for the nominal 0.5 oxidation conditions at 2800 K. Means and SDs refer to separate 220–230 ps trajectory averages. New-connection counts are instantaneous network descriptors averaged over time, not cumulative counts of reaction events.

Pristine C_60_ retained its initial C–C connections in all three trajectories: f_CC,ret_ = 1.0000 ± 0.0000. Its new-connection count was 0.191 ± 0.084 and S_CC_/N_C,0_ was 1.8569 ± 0.0036, consistent with little replacement of the original network in the sampled oxidation window.

V1 had f_CC,ret_ = 0.9558 ± 0.0145, 2.766 ± 0.711 new C–C connections and S_CC_/N_C,0_ = 1.7870 ± 0.0265. V2-adj had f_CC,ret_ = 0.9617 ± 0.0397, 4.597 ± 3.789 new connections and S_CC_/N_C,0_ = 1.8288 ± 0.0177. These values describe the sampled late-stage networks relative to the respective initial references.

At 2800 K, the sampled V2-adj oxidation trajectories preserve more initial connections and contain fewer replacement connections than V2-sep: f_CC,ret_ = 0.9617 ± 0.0397 versus 0.6231 ± 0.1279, and new C–C counts of 4.597 ± 3.789 versus 29.785 ± 9.706. These directions persist across the tested BO thresholds. The matched oxygen-free V2-adj controls also retain 0.9413 ± 0.0423 and contain 7.469 ± 3.551 new connections. Thus, the limited replacement of V2-adj is not unique to oxygen exposure. The atom-resolved analysis below examines where replacement connections occur and when selected pairs appear, while retaining the distinction between structural observations and a causal healing mechanism.

**Figure 4:**
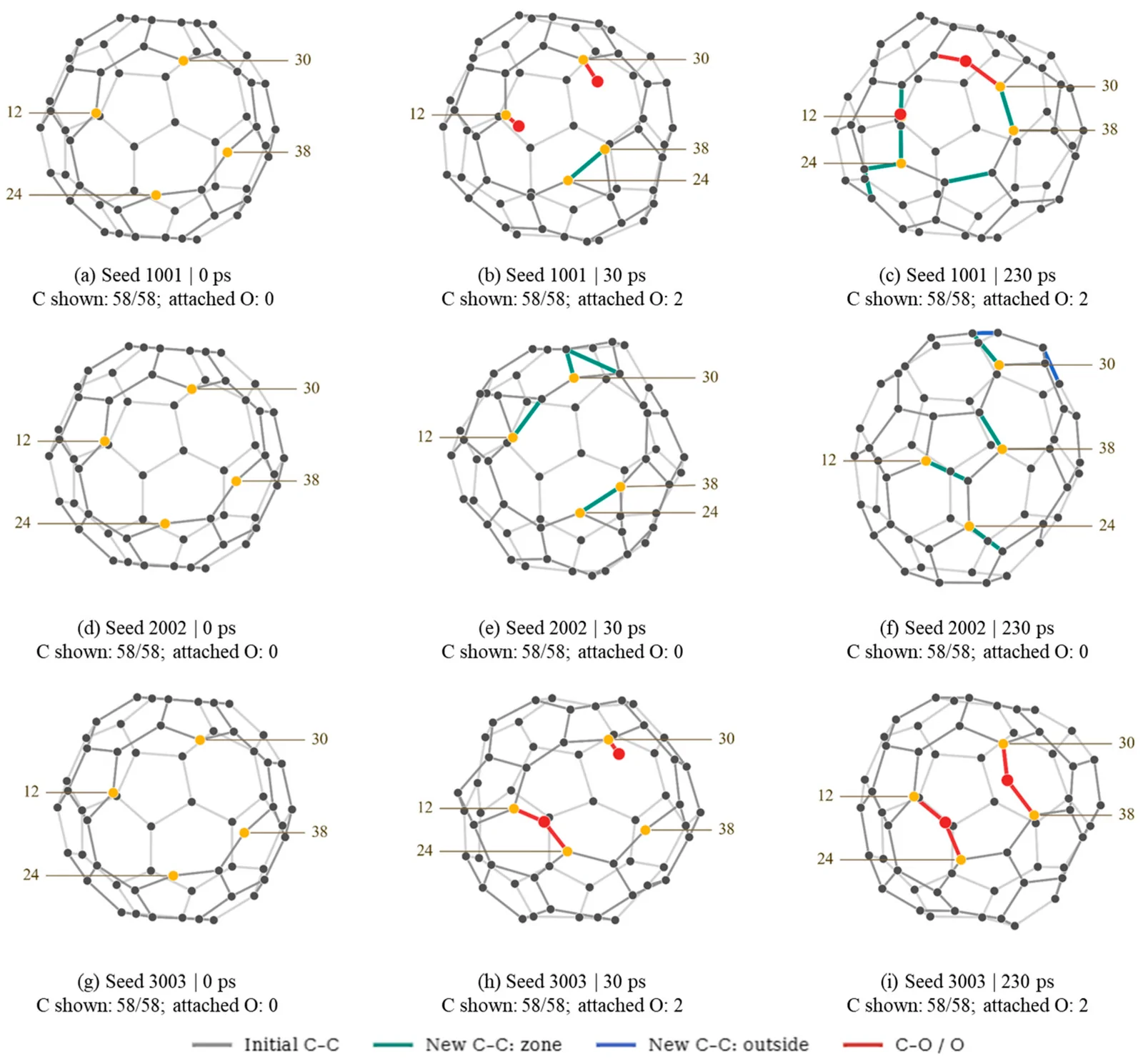
V2-adj structural snapshots for simulations with a production temperature of 2800 K. Rows: seeds 1001, 2002 and 3003; columns: total times of 0, 30 and 230 ps. The gold-highlighted atoms labeled 12, 24, 30 and 38 are the four initially undercoordinated carbon atoms adjacent to the vacancies; the labels denote fixed atom IDs retained throughout the trajectories.

The local analysis distinguishes the three V2-adj histories (Figure 4). At 2800 K, seed 2002 has no same-pair C–O episode lasting at least 1 ps and zero late-stage C–O bonding. Its new C26–C30 connection remains above BO = 0.30 from 22.5 to 230 ps, an observed span of 207.5 ps that begins during heating and is censored at the final frame. Further connections, including C24–C42, C21–C38 and C12–C33, start at 77.9–78.6 ps and remain until 230 ps. All four construction vacancy neighbor atoms have at least three C–C neighbors at this cutoff throughout the late window. Conversely, seeds 1001 and 3003 retain persistent C–O pairs beginning at total times 16.0 and 10.8 ps. Both therefore attach oxygen during heating, before the 2800 K production stage. Across the three seeds, late new zone-associated C–C counts are 7.188, 4.653 and 0.248; their late normalized C–O sums are 0.05988, 0 and 0.06238. These histories show different outcomes of reconstruction and attachment, but do not establish that 2800 K causes vacancy healing or a universal reduction in oxygen reactivity.

In the 2800 K late window, V2-adj has 4.030 ± 3.512 new zone-associated C–C connections and 0.568 ± 0.685 outside that construction zone, compared with 16.937 ± 4.926 and 12.848 ± 4.960 for V2-sep. Replacement extends beyond the initial vacancy neighbor identities more strongly in the sampled V2-sep networks. The zone sizes and initial undercoordination differ, so these counts are not equal-site-normalized measures of intrinsic vacancy reactivity. V2-adj oxygen-free controls also show long same-pair reconstruction episodes and zero late undercoordination of the four construction sites at the operational cutoff. Carbon neighbor counts do not measure chemical valence or prove closure of a geometric vacancy. The local counts and same-pair records support seed-dependent structural reconstruction, while oxygen encounters and thermally accessible routes remain alternative contributors to the lower sampled V2-adj mean.

The selected 230 ps V2-sep snapshots also show why stronger global C–O bonding is not equivalent to more oxygen retained on an intact cage. Their largest C–C-connected components contain 55, 53 and 56 of the initial 58 carbon atoms for seeds 1001, 2002 and 3003; the remaining carbon atoms form singleton C–C components at BO = 0.30. Carbon outside the largest component contributes 7.228 of 9.643, 13.214 of 13.214, and 4.887 of 7.736 to the respective unnormalized global C–O sums: 75.0%, 100% and 63.2%. Consequently, S_CO,norm_ includes oxygenated carbon outside the main network. These fixed-frame observations qualify the binding interpretation without providing a complete fragmentation history or CO/CO_2_ yield.

V2-sep showed f_CC,ret_ = 0.6231 ± 0.1279 and 29.785 ± 9.706 new connections, together with S_CC_/N_C,0_ = 1.7307 ± 0.0267. Relative to V2-adj, all three sampled V2-sep trajectories have lower initial retention and more new connections, although these global measurements do not identify where the rearrangements occur.

The number of new C–C connections in V2-sep, together with loss of initial connections and a lower total C–C bond-order sum, indicates connectivity replacement rather than a simple strengthening of the original cage. Global descriptors cannot locate these changes or establish a sequence of reactions at the two vacancy regions. Quantum chemical studies have demonstrated that carbon removal can modify fullerene ring structures [46,47], but they have not directly validated the present trajectories.

The mean absolute carbon charges were 0.01811 ± 0.00018 |e| for pristine C_60_, 0.03013 ± 0.00446 |e| for V1, 0.02435 ± 0.00512 |e| for V2-adj and 0.03992 ± 0.00427 |e| for V2-sep. These are trajectory-level uncertainties in the initial-carbon-population charge descriptor, not SDs over atoms.

The high C–O bond order sum, lower initial C–C retention and larger mean absolute carbon charge co-occur in V2-sep. This is an association among descriptors under the selected model, rather than proof that charge redistribution causes C–C dissociation or that reconstruction causes oxygen attachment.

The mean absolute charges are internal QEq descriptors. The nonzero pristine/O_2_ baseline and force-field dependence preclude treating them as measured charge transfer or proof of electronic localization. The oxygen-free carbon charges round to zero in the saved bond records; this contrast does not independently validate charge accuracy. ACKS2 or eReaxFF could affect both charges and trajectories because charges contribute to the forces. No charge method comparison or electronic structure charge benchmark was performed [34,37,38].

Figure 4 compares the V2-adj structures at total times of 0, 30 and 230 ps for all three independent seeds. The 30 ps snapshots mark the start of production at 2800 K. Seed 2002 shows reconstructed C–C connectivity without attached oxygen in these selected frames, whereas seeds 1001 and 3003 retain attached oxygen at 30 and 230 ps. All 58 carbon atoms belong to the largest C–C-connected component in these selected V2-adj frames. The contrasting histories are therefore visible without treating the absence of oxygen in a few snapshots as absence of all transient C–O encounters. The same-pair timings described above establish which connections endure; the snapshots alone do not establish geometric vacancy closure or a causal temperature-induced passivation mechanism. Production begins at 30 ps. Gray lines denote initial C–C connections; green and blue lines indicate new connections involving and outside the fixed construction vacancy neighbor zone, respectively. Oxygen atoms and C–O connections are red, and the four construction vacancy neighbor carbon atoms are gold and numbered. Connections are displayed at BO ≥ 0.30, with unbound oxygen omitted.

### 3.3. Effect of Oxygen Loading on Double-Vacancy Cages

**Figure 5:**
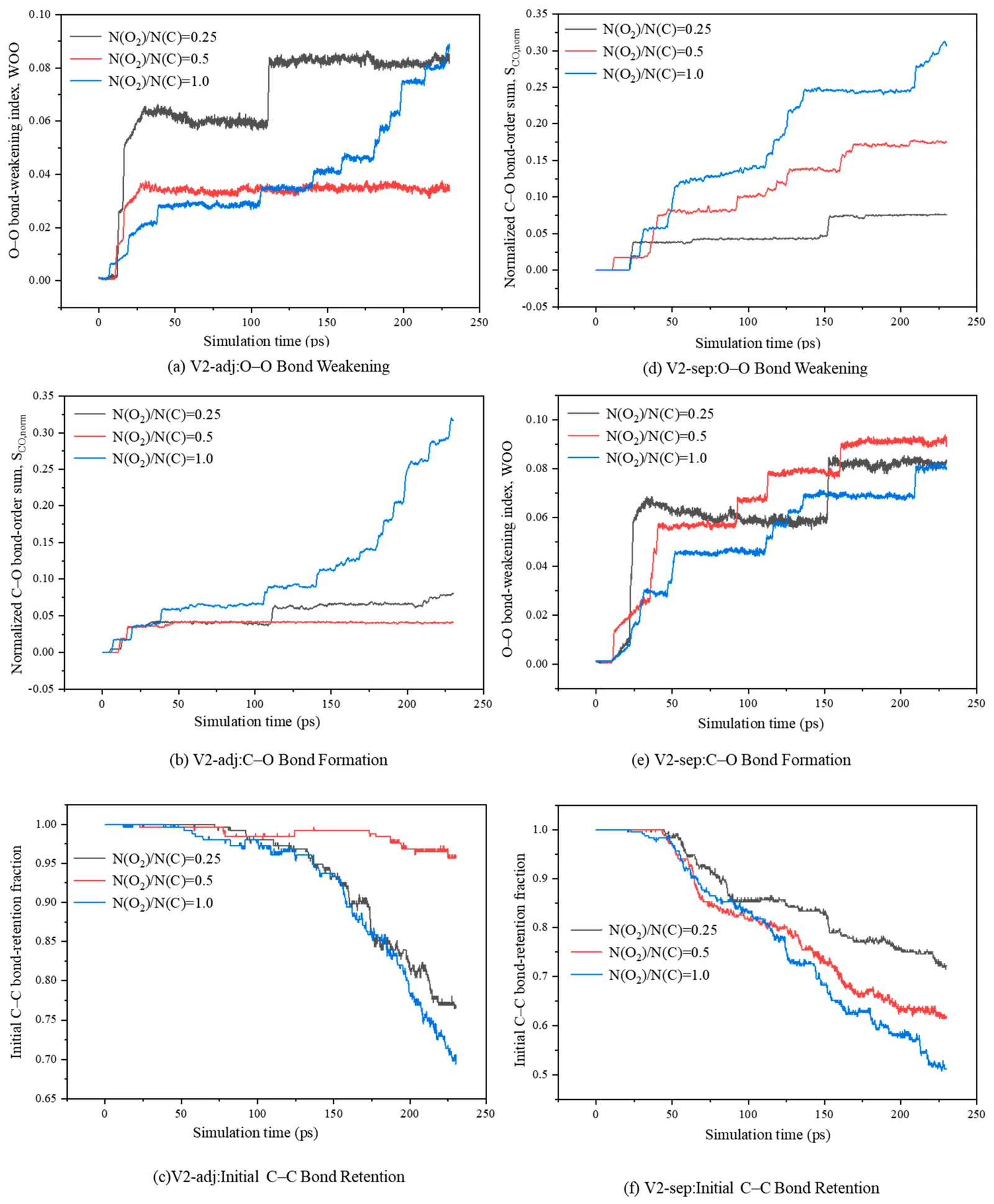
Effect of oxygen loading on the oxidation response of double-vacancy fullerene nanocages at 2800 K.

Figure 5 compares the two C_58_ configurations at nominal oxygen loadings of 0.25, 0.5 and 1.0 at 2800 K. The actual molecule/carbon ratios are 0.24138, 0.50000 and 1.00000, respectively. Table 2 provides the condition means and between-trajectory SDs.

For V2-adj, mean S_CO,norm_ values at nominal loadings of 0.25, 0.5 and 1.0 were 0.0779, 0.0408 and 0.2963; mean initial C–C retention was 0.7701, 0.9617 and 0.7142. The mean response is nonmonotonic, but its interpretation must consider the large condition-dependent SDs listed in Table 2.

The nominal 0.5 V2-adj condition combined the highest mean initial retention with the lowest mean C–O bonding. This is a sampled association, rather than evidence of a statistically established loading optimum. Vacancy closure and the temporal relation between rebonding and attachment are not demonstrated by these global descriptors; thermal reconstruction and oxygen encounter variability also contribute.

For V2-sep, mean S_CO,norm_ increased from 0.0762 to 0.1750 and 0.3015 across the three nominal loadings. The nominal 1.0 mean was about 3.96 times the nominal 0.25 mean. This describes greater sampled C–O bonding at higher oxygen population, not an elementary rate or an independently measured per-site reaction probability.

Mean V2-sep initial C–C retention decreased from 0.7289 to 0.6231 and 0.5174 across the loading series. More global C–O bonding thus coincided with more replacement of initial connectivity in the sampled means, although matched thermal controls show that oxygen is not the sole source of network rearrangement.

Mean V2-sep W_OO_ values were 0.0817, 0.0912 and 0.0810 across the nominal loading series. W_OO_ normalizes the weakening of initially introduced O–O pairs by their initial bond order sum, so it need not increase with the absolute oxygen population. It should be interpreted alongside C–O bonding and C–C retention.

The configuration contrast is most pronounced at nominal loading of 0.5, where mean S_CO,norm_ is 4.29 times greater for V2-sep than V2-adj. At nominal loading of 0.25, the means are similar (0.0779 and 0.0762). Thus, the observed contrast depends on oxygen loading as well as the chosen configurations; each condition has only three independent trajectories.

At nominal loading of 1.0, mean S_CO,norm_ is similar for V2-adj and V2-sep (0.2963 and 0.3015), while mean initial retention differs (0.7142 and 0.5174). The substantial seed variability in Table 2 limits the inference drawn from these mean differences.

Oxygen loading changes the opportunity for oxygen–carbon encounters, whereas the resulting network response also depends on the defect environment. Studies of defective carbon support the role of local coordination in oxygen adsorption [44,45], and quantum chemical calculations on vacancy-containing C_60_ show that carbon removal can change ring openings [46,47]. ReaxFF studies have also examined fullerene formation from porous carbon structures [48]. These comparisons motivate considering evolving carbon connectivity together with oxygen attachment, without treating pore accessibility or a specific adsorption pathway as directly established by the present global descriptors.

### 3.4. Integrated Interpretation and Limits of Applicability

Table 2 and Table 3 distinguish configuration-dependent C–O bonding from thermal connectivity replacement. V2-sep combines more C–O bonding and more replacement of initial C–C connections than V2-adj at nominal loading of 0.5 and 2800 K. The matched controls show that substantial reconstruction can also occur without oxygen, and that the effect of oxygen on retention is configuration dependent. These results concern the evolving network descriptors, not atom-resolved reaction sequences or oxidation probabilities.

An additive two-V1 benchmark corrects for the different carbon normalization: S_CO,norm,add_ = 2 × (59/58) × 0.0933445 = 0.1899077. V2-sep has S_CO,norm_ = 0.1750063, giving a contrast of −0.0149014 (7.85% below the benchmark). For independent V1 and V2-sep samples, the propagated SEM of this contrast is [s_sep_^2^/3 + (2 × 59/58)^2^ s_V1_^2^/3]^½^ = 0.0536355. The mean departure is therefore small relative to its sampling uncertainty; it does not establish suppression of oxidation at each vacancy. The benchmark is a descriptive sum, not a separately simulated two-defect system. Both regions share one finite cage and oxygen environment, and the global additive comparison does not determine a causal oxidation probability for either vacancy.

At 2800 K, oxygen-free V1 shows f_CC,ret_ = 0.4765 ± 0.1449 and 48.010 ± 12.580 new C–C connections, whereas the oxidation trajectories show 0.9558 ± 0.0145 and 2.766 ± 0.711 (Table 3). The sampled initial V1 connectivity is therefore substantially more preserved in the oxygen-containing condition; this is compatible with oxygen altering available reconstruction routes but does not establish a specific passivation mechanism. V2-adj has relatively high retention in both environments: 0.9413 ± 0.0423 without oxygen and 0.9617 ± 0.0397 with oxygen. For V2-sep, oxygen-free retention is 0.7388 ± 0.2091 versus 0.6231 ± 0.1279 with oxygen, and new connections are 26.277 ± 16.855 versus 29.785 ± 9.706; the broad overlapping seed ranges preclude a strong directional attribution to oxygen from these values alone.

Thermal replacement in defective cages is also evident at lower temperatures. For example, oxygen-free V1 retention decreases from 0.9734 ± 0.0461 at 1600 K to 0.7731 ± 0.0484 at 2200 K. These controls rule out a general interpretation of every lost or new C–C connection as an oxidation event. Each retention fraction uses that trajectory’s initial network; loss of an initial connection need not imply fragmentation or carbon removal. Three independent seeds per environment still give limited statistical confidence.

Both environments use 200 ps production and the same 220–230 ps statistics window, with independent initial-velocity seeds. Initial reference pairs are defined separately for each trajectory. O_2_ labels the nominal 0.5 oxidation condition; No O_2_ labels the matched oxygen-free control.

The principal late-stage C_58_ contrast is preserved when the BO threshold increases from 0.30 to 0.40. V2-adj retention changes from 0.96168 to 0.96133 and new connections from 4.597 to 4.188; V2-sep retention changes from 0.62309 to 0.62168 and new connections from 29.785 to 29.294. Their normalized C–O sums remain 0.04075 and 0.17501. Across all 16 oxidation conditions, the maximum absolute changes in condition means are 0.00033424 for normalized C–O bonding, 0.00141443 for retention and 0.64356 for new connections. Rare weak connections can nevertheless have large relative changes. These numerical comparisons delimit the sensitivity of the retained descriptors within the tested cutoff range.

Persistence tests span 0.5, 1.0 and 2.0 ps using actual elapsed time. At BO = 0.30, the same 37 of 48 oxidation trajectories contain a persistent C–O connection state during production at all three criteria. Some first-state times change, however; across the tested BO thresholds, the largest change in observed C–O first-state time is 10.7 ps. Short-lived new C–C connection states are more sensitive, and the corresponding first-state times can change substantially. The original ten-frame implementation spanned only 0.9 ps at 0.1 ps sampling; it has been corrected. These results support robustness of the principal late-stage descriptors within the tested cutoff range, rather than universal insensitivity of event times or atom pair lifetimes. These aggregate connection existence states need not track one identical atom pair; the fixed-pair analysis in Section 3.2 uses the stricter same-pair definition.

Experimental C_60_ powder oxidation has been described using a contracting sphere model at an agglomerate interface [40], while oxidation of fullerite by interstitial oxygen involves a condensed-phase environment [41]. In situ soot microscopy also shows nanostructure-dependent spatial oxidation behavior. These studies provide qualitative context for the roles of accessible sites and evolving carbon structure; none directly compares the two isolated C_58_ vacancy configurations. Oxygen-free ReaxFF fullerene fragmentation further shows why C–C reconstruction alone is not a specific marker of oxidation. Agreement in absolute rates, onset temperatures or the present topology ranking is not claimed.

The initial C_60_ radius is 3.5188 Å (0.3519 nm), corresponding to an approximate curvature scale 1/R = 2.84 nm^−1^ and a diameter of 0.704 nm. This strongly curved cage omits the size, graphitic layers, surfaces and aggregation of soot primary particles. No larger fullerene, radius series or graphene limit was simulated; size, cage connectivity and curvature cannot be separated by the present comparison. Extrapolation to zero curvature or real soot oxidation therefore remains untested. A future size series would need consistent defect definitions and oxygen exposure, paired with a planar reference, rather than an extrapolation of the C_60_ values alone.

The observations remain conditional on the selected ReaxFF/QEq model. The matched controls, cutoff tests and atom-resolved reconstruction records strengthen the assessment of thermal and local network changes but do not validate defective-site reaction energies or charge accuracy. The structural evidence shows different sampled reconstruction and attachment histories; it does not prove geometric vacancy closure or that reconstruction uniquely causes suppression of oxygen attachment. Additional vacancy isomers, electronic structure benchmarks, charge model comparisons and lower-temperature trajectories may change the response.

## 4. Conclusions

ReaxFF/QEq simulations compared early oxygen attachment and carbon network reconstruction for pristine C_60_, V1 and two specific C_58_ configurations at 1600–2800 K. Three independent trajectories per condition, matched 200 ps oxygen-free controls and bond order/persistence tests support a conditional comparison of these selected networks.

Pristine C_60_ showed very little late-stage C–O bonding. At 2800 K and nominal loading of 0.5, S_CO,norm_ was 0.09334 ± 0.03989 for V1, 0.04075 ± 0.03532 for V2-adj and 0.17501 ± 0.04520 for V2-sep. The lower V2-adj mean at 2800 K accompanies substantial seed variation and does not establish a temperature-induced suppression mechanism.

V2-adj retained 0.9617 ± 0.0397 of its initial C–C connections and contained 4.597 ± 3.789 new connections, compared with 0.6231 ± 0.1279 and 29.785 ± 9.706 for V2-sep. The direction of this difference persisted over BO = 0.30–0.40. It concerns the two tested C_58_ configurations, whose initial undercoordination differs; it does not establish a general law for adjacent and separated vacancies. Atom-resolved records further show replacement beyond the construction vacancy neighbor zone in V2-sep and distinct seed-dependent V2-adj histories; some enduring reconstruction and attachment episodes already begin during heating. In the three selected final V2-sep snapshots, the largest C–C-connected component contains 53–56 of the initial 58 carbon atoms, and global C–O bonding includes contributions outside it.

V2-sep was 7.85% below the carbon-normalized additive two-V1 mean, but the contrast (−0.01490) was smaller than its propagated SEM (0.05364). This descriptive departure does not establish a lower oxidation probability per vacancy.

Higher nominal oxygen loading increased the sampled mean C–O bonding of V2-sep from 0.0762 to 0.3015. Matched controls also showed extensive thermal reconstruction, particularly for V1. Consequently, oxygen participation changes the network response in a configuration-dependent manner; connectivity replacement alone is not evidence of oxygen-induced fragmentation.

The findings are limited to the selected configurations, three trajectories per condition, observation window and ReaxFF/QEq parametrization. They establish neither defective-site energetic accuracy nor a unique local healing mechanism. Event times and short-lived connections remain definition dependent. Larger cages, other isomers, alternative charge models and lower-temperature conditions require separate assessment; the present early-stage descriptors cannot be quantitatively extrapolated to real soot oxidation kinetics.

## Figures and Tables

**Table 1:** Reactive molecular dynamics simulation matrix.

Simulation Group	Nanocage Structures	Temperature	Nominal O_2_ Loading	Random Seeds	Production Time
Principal oxidation matrix	Pristine, V1, V2-adj, V2-sep	1600, 2200 and 2800 K	0.5	1001, 2002 and 3003	200 ps
Oxygen-free controls	Pristine, V1, V2-adj, V2-sep	1600, 2200 and 2800 K	0	9001, 9002 and 9003	200 ps
Oxygen-loading sensitivity	V2-adj and V2-sep	2800 K	0.25 and 1.0	1001, 2002 and 3003	200 ps

**Table 2:** Late-stage oxidation descriptors as mean ± sample SD across three independent trajectories.

Cage	T (K)	λ_nom_	W_OO_	S_CO,norm_	f_CC,ret_	N_new_	S_CC_/N_C,0_	⟨|q_C_|⟩
C_60_	1600	0.5	0.0068 ± 0.0003	0.00000 ± 0.00000	1.0000 ± 0.0000	0.007 ± 0.006	1.8835 ± 0.0018	0.0168 ± 0.0001
V1	1600	0.5	0.0382 ± 0.0003	0.05709 ± 0.00095	1.0000 ± 0.0000	0.033 ± 0.041	1.8457 ± 0.0001	0.0249 ± 0.0003
V2-adj	1600	0.5	0.0408 ± 0.0013	0.06142 ± 0.00320	1.0000 ± 0.0000	0.234 ± 0.046	1.8425 ± 0.0020	0.0268 ± 0.0010
V2-sep	1600	0.5	0.0511 ± 0.0199	0.07622 ± 0.03256	0.9602 ± 0.0345	4.739 ± 3.271	1.8267 ± 0.0203	0.0278 ± 0.0040
C_60_	2200	0.5	0.0101 ± 0.0015	0.00000 ± 0.00000	1.0000 ± 0.0000	0.040 ± 0.026	1.8708 ± 0.0010	0.0174 ± 0.0003
V1	2200	0.5	0.0426 ± 0.0013	0.05700 ± 0.00033	1.0000 ± 0.0000	0.092 ± 0.021	1.8369 ± 0.0025	0.0254 ± 0.0002
V2-adj	2200	0.5	0.0426 ± 0.0005	0.05987 ± 0.00509	0.9920 ± 0.0139	1.287 ± 1.749	1.8286 ± 0.0014	0.0267 ± 0.0017
V2-sep	2200	0.5	0.0547 ± 0.0204	0.07774 ± 0.03235	0.8613 ± 0.1316	12.713 ± 11.652	1.8226 ± 0.0195	0.0288 ± 0.0041
C_60_	2800	0.5	0.0126 ± 0.0020	0.00002 ± 0.00003	1.0000 ± 0.0000	0.191 ± 0.084	1.8569 ± 0.0036	0.0181 ± 0.0002
V1	2800	0.5	0.0568 ± 0.0204	0.09334 ± 0.03989	0.9558 ± 0.0145	2.766 ± 0.711	1.7870 ± 0.0265	0.0301 ± 0.0045
V2-adj	2800	0.5	0.0350 ± 0.0194	0.04075 ± 0.03532	0.9617 ± 0.0397	4.597 ± 3.789	1.8288 ± 0.0177	0.0244 ± 0.0051
V2-sep	2800	0.5	0.0912 ± 0.0192	0.17501 ± 0.04520	0.6231 ± 0.1279	29.785 ± 9.706	1.7307 ± 0.0267	0.0399 ± 0.0043
V2-adj	2800	0.25	0.0836 ± 0.0007	0.07793 ± 0.00797	0.7701 ± 0.1388	19.901 ± 12.588	1.8083 ± 0.0147	0.0243 ± 0.0006
V2-sep	2800	0.25	0.0817 ± 0.0717	0.07615 ± 0.07284	0.7289 ± 0.0731	23.881 ± 5.580	1.8012 ± 0.0431	0.0239 ± 0.0080
V2-adj	2800	1.0	0.0822 ± 0.0772	0.29632 ± 0.32776	0.7142 ± 0.3321	14.974 ± 14.619	1.6524 ± 0.2344	0.0593 ± 0.0414
V2-sep	2800	1.0	0.0810 ± 0.0004	0.30155 ± 0.01688	0.5174 ± 0.0798	32.937 ± 6.309	1.6293 ± 0.0329	0.0593 ± 0.0008

All values were recalculated from unsmoothed 220–230 ps averages computed separately for each trajectory (101 frames per trajectory). λ_nom_ is the nominal loading label; exact O_2_ counts and actual ratios are given in Section 2.3. W_OO_, S_CO,norm_, f_CC,ret_, N_new_, S_CC_/N_C,0_ and ⟨|q_C_|⟩ denote initial O–O weakening, normalized C–O bonding, initial C–C retention, new C–C connection count, normalized C–C bonding and mean absolute carbon charge (|e|). New-connection counts are time averages, not cumulative events. SD is between independent trajectories. Each condition contains three independent trajectories.

**Table 3:** Matched oxidation and oxygen-free carbon network descriptors at 2800 K (mean ± SD, *n* = 3).

Cage	f_CC,ret_ O_2_	f_CC,ret_ No O_2_	N_new_ O_2_	N_new_ No O_2_	S_CC_/N_C,0_ O_2_	S_CC_/N_C,0_ No O_2_
C_60_	1.0000 ± 0.0000	1.0000 ± 0.0000	0.191 ± 0.084	0.248 ± 0.010	1.8569 ± 0.0036	1.8547 ± 0.0028
V1	0.9558 ± 0.0145	0.4765 ± 0.1449	2.766 ± 0.711	48.010 ± 12.580	1.7870 ± 0.0265	1.8576 ± 0.0040
V2-adj	0.9617 ± 0.0397	0.9413 ± 0.0423	4.597 ± 3.789	7.469 ± 3.551	1.8288 ± 0.0177	1.8532 ± 0.0050
V2-sep	0.6231 ± 0.1279	0.7388 ± 0.2091	29.785 ± 9.706	26.277 ± 16.855	1.7307 ± 0.0267	1.8671 ± 0.0192

## Data Availability

The original contributions presented in this study are included in the article. Further inquiries can be directed to the corresponding author.
